# Can instrumentation kinematics affect postoperative pain and substance P levels? A randomized controlled trial

**DOI:** 10.1186/s12903-024-03882-x

**Published:** 2024-01-17

**Authors:** Salma Talaat Abdel-Baset, Sarah Hossam Fahmy, Maram Farouk Obeid

**Affiliations:** Department of Endodontics, Faculty of Dentistry, Ain Shams University, Cairo, Egypt

**Keywords:** Substance P, Continuous rotation, Reciprocation, Postoperative pain, Symptomatic apical periodontitis

## Abstract

**Background:**

This study aimed to assess the influence of continuous rotation and reciprocation kinematics on postoperative pain (POP) levels and substance P (SP) levels in patients diagnosed with irreversible pulpitis and symptomatic apical periodontitis (SAP).

**Materials and subjects:**

A total of twenty patients were randomly assigned into two groups: Continuous Rotation Group (CRG) (*n* = 10), subjected to mechanical preparation with the EdgeEndox7 rotary system (Albuquerque, NM, USA), and Reciprocation Group (RG) (*n* = 10), treated with the EdgeOne Fire reciprocating system (Albuquerque, NM, USA). Apical fluid (AF) samples were collected, and SP levels were quantified through radioimmunoassay. POP was assessed using the Numerical Rating Scale (NRS) at various time intervals (preoperatively, 6 h, 12 h, 24 h, 48 h, and 72 h). Data were statistically analyzed utilizing the independent t-test, Mann-Whitney U test, Friedman’s test, and Nemenyi post hoc test.

**Results:**

There was a significant increase in SP levels in the reciprocating group compared to the continuous rotation group (*P* ≤ 0.05). Additionally, patients in the reciprocating group reported significantly higher POP levels (*P* ≤ 0.05) at all measured intervals (6 h, 12 h, 24 h, and 48 h), with both groups exhibiting similar pain level reductions at the 72-hour mark.

**Conclusion:**

Our findings suggest that continuous rotation kinematics in root canal preparation leads to a considerable reduction in SP expression and POP.

**Trial registration:**

The study protocol was retrospectively registered on the www.clinicaltrials.gov database (NCT06081335) at (13/10/2023) after the approval of the Ethics Committee, Faculty of Dentistry, Ain Shams University (FDASU-RecIM012135).

## Background

The success of root canal treatment depends greatly on removing pulp tissue remnants, microorganisms, and microbial toxins from the root canal system [1], which is accomplished by chemomechanical preparation of the root canal. Traditionally, stainless steel hand instruments were used for shaping root canals but iatrogenic errors such as canal transportation and ledges were inevitable due to the low flexibility of such instruments [2]. With the evolution of nickel-titanium engine-driven files, the game has been changed as they have played a great role in minimizing these errors and increasing preparation safety [3].

Over time, manufacturers modified file design and machining processes while others focused on metallurgy, and heat treatment [4]. Maximizing flexibility and minimizing fracture are the main goals of these exclusive modifications [5]. Recently, Henry Schein Dental introduced the EdgeEndoX7 system (EdgeEndo, Albuquerque, NM, USA) with “Canal Contouring Technology”, attributed to the unique FireWire™ heat-treatment method. They claimed that this technology raises the files’ flexibility and lessens the typical restoring force experienced with other NiTi files [6]. These files feature a triangular cross-section, a constant 0.04 taper, and a changeable helix angle [7].

To achieve the same goals of reducing torsional loads and boosting fracture resistance, a recent suggestion was that instrumentation kinematics can be changed from continuous rotation to alternate clockwise, and counterclockwise reciprocating motion [8]. Using FireWire™ technology, Henry Schein Dental revealed the EdgeOne Fire reciprocating system (EdgeEndo, Albuquerque, New Mexico, USA) with a parallelogram cross-section having two cutting edges and an off-center design [9]. Cutting and releasing angles are used in a reciprocating manner; with an exchange in the direction of rotation [10].

The inherent propensity of extruding bacteria, pulp tissue, dentinal chips, and irrigants peri-apically during root canal preparation is inevitable [11] and much evidence highlights the intimate relation between instrument motion and debris extrusion [12, 13]. Unfortunately, one of the most important issues that arises from these extrusions is POP, which is an annoying event for both patients and dentists [14, 15].

The Numerical Rating Scale (NRS), Verbal Rating Scale (VRS), and Visual Analog Scale (VAS) are some of the tools used to monitor POP [16]. Unfortunately, due to patients’ subjectivity, patients as a factor have a significant effect on these metrics [16]. As a result, several investigations [17–19] found a connection between POP levels and the production of neuropeptides by sensory pulpal neurons in response to harmful stimuli. As an illustration, afferent fibers (nociceptors) generate substance P (SP) which triggers neurogenic inflammation and irreversible pulpitis [17]. According to Arun N and Ramesh S [19], SP can be used to corroborate and contrast POP.

Nonetheless, research about the relationship between kinematics and POP is still inconsistent and occasionally contradictory [12, 13, 15] and to reinforce evidence-based results, randomized clinical trials are needed to evaluate clinical outcomes. Thus, the rationale for this study was to assess the incidence of POP and SP levels after using the EdgeEndox7 and EdgeOne Fire systems with different kinematics in patients with irreversible pulpitis in mandibular second premolars with symptomatic apical periodontitis (SAP). The null hypothesis presupposes that when either preparatory kinematics was used, there was no difference in SP levels or POP in patients with SAP.

## Participants and methods

### Study design and setting

**Fig. 1:**
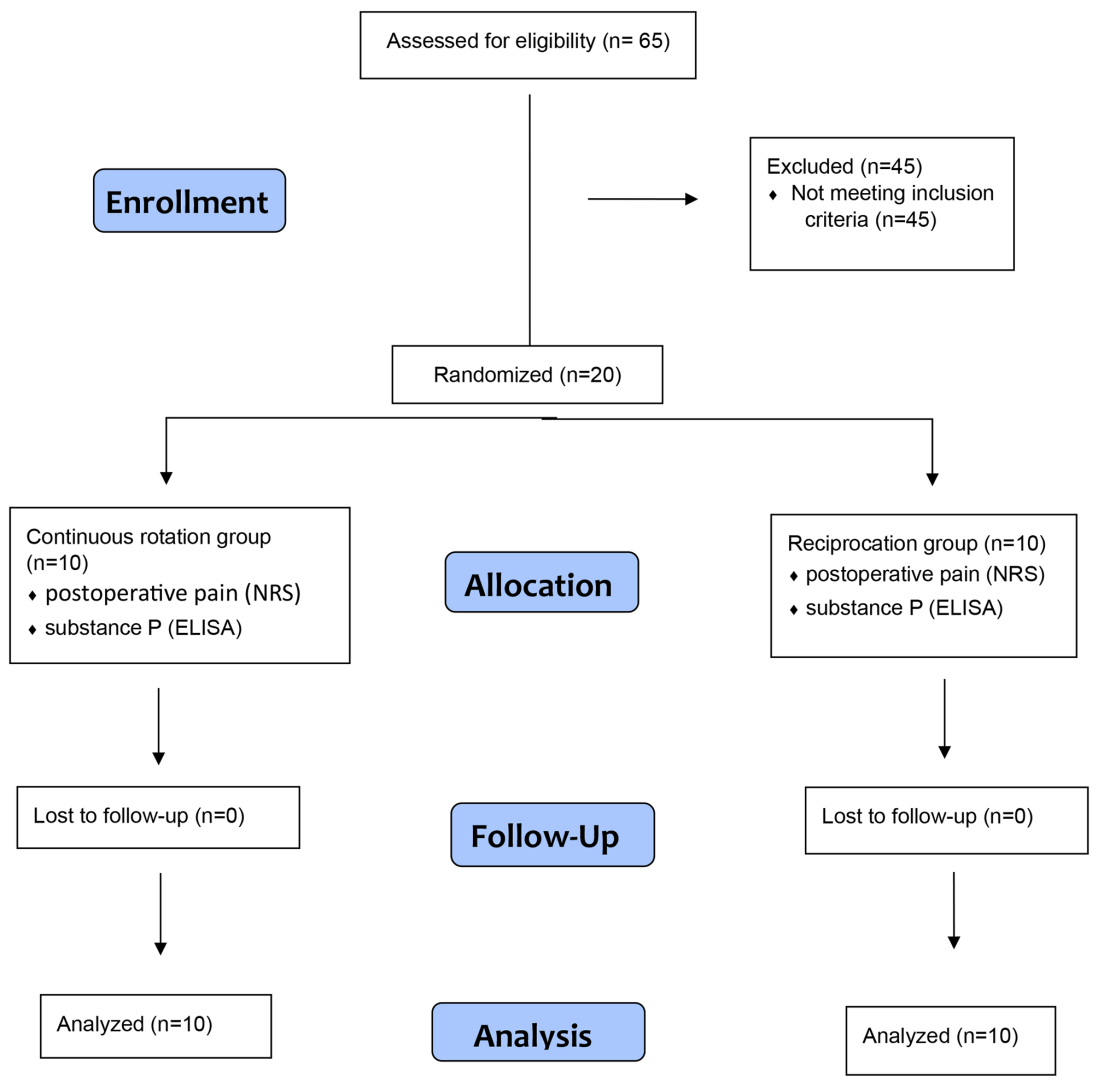
Consolidated standards of reporting trials flow diagram of the study

This is a single-blinded, double-arm, randomized controlled clinical trial that was designed and reported by adhering to the Consolidated Standards of Reporting Trials statement CONSORT [20]. A flow diagram representing the Consolidated Standards of Reporting Trials of the study is presented in (Fig. 1). The clinical trial only started after obtaining the approval of the Ethical Committee Faculty of Dentistry, Ain Shams University (FDASU-RecIM012135).

The study population was recruited from the outpatient endodontic clinic at the Faculty of Dentistry, Ain Shams University. All applicants signed a written consent form after a detailed description of the study’s aim, methods, advantages, and possible hazards.

### Sample size calculation and power analysis

A power analysis was designed to have adequate power to apply a two-sided statistical test of the null hypothesis that there is no difference between tested groups. By adopting an alpha level of (0.05) and a beta of (0.2), i.e., power = 80% and an effect size (d) of (1.25) calculated based on the results of Caviedes-Bucheli J [21]; the predicted sample size (n) was found to be a total of cases (i.e., 10 cases per group). Sample size calculation was performed using G*Power version 3.1.9.7 2.

### Eligibility criteria

The inclusion criteria encompassed healthy individuals aged between 20 and 50 years, possessing single-canaled mandibular second premolars with complete root formation. Additionally, patients were required to have a pulpal diagnosis of symptomatic irreversible pulpitis and a periapical diagnosis of symptomatic apical periodontitis, without a visible periapical radiolucent area. Furthermore, patients needed to report preoperative pain levels above 4 on the NRS to ensure standardization.

Exclusion criteria included the presence of systemic diseases or allergic reactions, current antibiotic or anti-inflammatory medication use, analgesic intake 12 h before treatment, radiographically untraceable canals or excessively curved roots, teeth with open apices, severe periodontal disease (either generalized or localized to the tooth in question), and the absence of bleeding in the pulp chamber upon access cavity preparation.

### Randomization

Patients who met the eligibility criteria were randomly assigned to two comparative parallel groups each (*n* = 10). CRG: received mechanical preparation with the EdgeEndox7 rotary system, and RG: treated with the EdgeOne Fire reciprocating system. This allocation was concealed in folded, numbered papers and placed in tightly sealed envelopes containing the patient’s coding and the random sequence was generated by computer software.

### Treatment protocol and interventions

#### Diagnosis

The treatment was carried out by a single operator where demographic data were collected, and proper diagnosis was accomplished. All teeth in the research reacted exaggeratedly to cold pulp sensibility tests (Endo- Frost, Coltene- Whaledent, Switzerland), and copious bleeding of the pulp was visible upon gaining access to the pulp chamber. The periapical diagnosis was confirmed by a positive response to palpation and percussion. At the beginning of the first visit, the NRS was explained to each patient, and they were asked to rate their pain levels from 0 (no pain) to 10 (worst possible pain).

#### Access preparation

All patients were anesthetized using 4% ARTINIBSA solution with 1:100,000 epinephrine (Inibsa, Spain), and then the operative field was isolated using a rubber dam. Access cavity preparation was performed under magnification.

#### Working length determination

Using a #15 K-file (Mani, Japan) with a root ZX apex locator (J Morita, Tokyo, Japan), working length determination was performed and followed by radiographic confirmation. Pulp extirpation and canal patency were gained to the working length followed by 2.5% sodium hypochlorite irrigation.

#### Apical fluid (AF) sample collection

To collect the first apical fluid sample (S1), the canal was dried adequately, and then size a (15.02) paper point (DiaDent Group, Seoul, Korea) was advanced 1–2 mm beyond the apex, held in place for 60 s, placed in an Eppendorf tube with 1 mL phosphate-buffered saline (PBS) (pH 7.4) and refrigerated at 10 °C for later examination [22].

#### Canal preparation

According to the assigned group:

##### CRG

The canals were enlarged apically to a size of 45 according to the manufacturer’s instructions. A Motopex endodontic motor (Woodpecker, Guilian, China) was used at 350 RPM and 3 Ncm. Coronal flaring was performed using file #25.12, and mechanical preparation was performed starting with #20.04 until #45.04 without skipping a file (#25.12, #20.04, #25.04, #30.04, #35.04, #40.04, and #45.04).

##### RG

The Motopex endodontic motor (Woodpecker, Guilian, China) was set in reciprocating mode with 150 degrees counterclockwise (CCW), and 30 degrees clockwise (CW) at 300 RPM and 2 Ncm, and then canal preparation was completed using the file sequence (#25.12, #20.04, #25.04, #35.04, and #45.04).

All canals were irrigated conventionally with 2 mL of 2.5% NaOcl between files. A final rinse with 5 mL of 2.5% NaOcl was applied for 1 min, followed by 5 mL of 17% EDTA (Meta Biomed, Korea) for 1 min. Irrigants were delivered by a two-sided–vented needle, gauge size 25.

#### Temporary restoration

Canals were dried, and the access cavity was temporarily restored with the glass ionomer Fuji IX (Tokyo, Japan). Patients were given the NRS sheet and were asked to mark their pain levels at 6 h, 12 h, 24 h, 48 h, and 72 h.

**The second visit** was scheduled after 5 days. Proper tooth isolation as described earlier was performed, temporary filling was removed, and a subsequent sample (S2) was extracted from the apical fluid using the same procedure as the first sample collection.

#### Root canal filling

After irrigation with 2.5% sodium hypochlorite and concluding with a final flush of 17% EDTA with saline irrigation in between. The root canals were meticulously dried using absorbent paper points. Subsequently, a master cone sized at #45 was carefully inserted into the canals, and a radiograph was taken to confirm the appropriate working length. The process then proceeded to obturation, which was accomplished through warm vertical compaction employing the Woodpecker obturation system Fi-P and Fi-G (Guilin Woodpecker Inc., China).

### Biochemical examination

The collected paper points were cut and diluted in 600 mL of PBS. The materials were then centrifuged at 10,000 rpm for 5 min. Using an enzyme-linked immunosorbent assay (ELISA) kit, substance P was quantified according to the manufacturer’s instructions. The lowest limit of SP detection with this kit was 3.9 pg mL ^− 1^. Each sample’s absorbency was determined at wavelengths between 420 and 450 nm in a microplate reader (SpectraMax Plus 384, USA). A standard curve was used to calculate the SP concentration in each sample.

### Statistical analysis

Numerical data are presented as the mean, standard deviation (SD), median, and interquartile range (IQR) values. They were explored for normality by checking the data distribution, and by using Shapiro-Wilk’s test. Normally distributed data (substance P) were analyzed using independent and paired t-tests for intergroup and intragroup comparisons, respectively. Nonparametric data (NRS) were analyzed using the Mann-Whitney U test for intergroup comparisons and the signed-rank test for intragroup comparisons. The significance level was set at *p* < 0.05. Statistical analysis was performed with R statistical analysis software version 4.3.1 for Windows^1^.

## Results

The study was conducted on 20 cases that were randomly and equally allocated to each of the tested groups (i.e., 10 cases each). No statistically significant difference was found between groups regarding demographic data which are presented in Table 1. None of the patients took medication for pain postoperatively.

**Table 1:** Intergroup comparisons and summary statistics for demographic data

Parameter		Reciprocation (RG)	Continuous rotation (CRG)	*P* value
Sex [n (%)]	Male	6 (60.0%)	6 (60.0%)	**1**
	Female	4 (40.0%)	4 (40.0%)	
Age (Mean ± SD)		38.20 ± 5.11	40.20 ± 6.45	**0.452**

*; significant (*P* < 0.05) ns; nonsignificant (*P* > 0.05)

Regarding POP analyzed from the NRS values (**mean ± SD**): Intergroup comparison showed that RG (8.50 ± 1.35) had a higher value than CRG (7.80 ± 1.32) yet, the difference was not statistically significant (*P* = 0.261), while in the other intervals RG had a significantly higher value than CRG (*P* < 0.05), except at 72 h at which both recorded zero. Intragroup results showed a significant difference between values measured at different intervals (*P* < 0.01) as shown in Table 2.

**Table 2:** Inter, and intragroup comparisons, and mean and standard deviation values of postoperative pain (NRS)

Interval	Postoperative pain (NRS) (mean ± SD)		*P* value
	Reciprocation (RG)	Continuous rotation (CRG)	
Preoperative	8.50 ± 1.35^A^	7.80 ± 1.32^A^	**0.261**
6 h	5.50 ± 1.96^AB^	3.00 ± 1.25^AB^	0.007*
12 h	4.10 ± 1.45^BC^	2.00 ± 1.33^BC^	0.007*
24 h	2.60 ± 0.97^BC^	1.50 ± 1.43^BC^	0.043*
48 h	1.60 ± 1.07^C^	0.70 ± 1.06^C^	0.049*
72 h	0.00 ± 0.00	0.00 ± 0.00	**NA**
*P* value	< 0.001*	< 0.001*	

Values with different superscript letters within the same **vertical column** are significantly different *; significant (*P* < 0.05) ns; nonsignificant (*P* > 0.05)

Regarding SP levels (pg/ml) analyzed by ELISA tests, within both groups, there was a significant reduction in substance P levels at S2 (*P* < 0.001) (Table 3). Moreover, the mean difference between groups (S1-S2) was also significantly different (*P* < 0.001).

**Table 3:** Intergroup and intragroup comparisons, and summary statistics of SP level (pg/ml)

Samples	Substance P level (pg/ml) (mean ± SD)		*P* value
	Reciprocation RG	Continuous rotation CRG	
S1	112.97 ± 67.80	41.37 ± 19.89	
S2	71.87 ± 47.81	23.10 ± 14.49	
Mean difference (S1-S2) (pg/ml)	41.00	18.27	< 0.001*
*P* value	< 0.001*	< 0.001*	

*; significant (*P* < 0.05)

## Discussion

The current randomized clinical trial (RCT) intended to determine the effects of various instrumentation kinematics on the levels of SP as a primary outcome and POP as a secondary outcome following root canal treatment of single-rooted mandibular second premolars with SAP. The study’s strength is that RCTs offer the greatest degree of evidence since they reduce systematic error (bias) and confounding variables [23]. As a result, they present the most accurate and trustworthy data on an intervention’s efficacy.

Preoperative pain has been proven to be one of the best predictors of postoperative pain [24]. Additionally, in symptomatic irreversible pulpitis, hyperalgesia, and allodynia (in peripheral and central pathways) continue even after root canal therapy [25]. Therefore, to ensure the presence of inflammation in and around the root, single-root mandibular second premolars with irreversible pulpitis and symptomatic apical periodontitis were the chosen conditions for inclusion.

Only patients who did not take any pain-modulating medications 12 h before the visit were allowed to participate in the study to abolish the influence of pretreatment analgesics on pain analysis [26]. Pain sensations with cold application and bleeding from the pulp during access cavity preparation were used to guarantee pulp vitality [27]. Pain on percussion was used to diagnose apical periodontitis [28].

The two groups exhibited no significant distinctions in terms of gender, age, or preoperative pain characteristics. Therefore, it is inferred that these variables did not impact the outcomes of the study. Some studies found no statistically significant difference between men and women in terms of postoperative pain severity [29, 30] which is in contrast to other research [22, 31].

Rotary Ni-Ti systems from the same manufacturer were selected for this study to ensure that they underwent a similar heat treatment process (FireWire™ treatment). This involves a combination of heat treatment and cryogenic applications to enhance file flexibility and minimize restoring force, as per the manufacturer’s claim [7, 32]. however, they differed in their kinematics where the EdgeEndo x7 files were employed in a continuous rotation motion, while the EdgeOne Fire files were utilized in a reciprocating motion with parameters set at 150 degrees CCW, and 30 degrees CW [9, 10].

The numerical rating scale (NRS) was employed for subjective pain assessment because it offers advantages in terms of its simplicity and ease of use [33], while SP levels were quantitatively measured as an objective assessment from apical fluid samples using the ELISA test which is the gold standard for quantifying inflammatory mediators and neuropeptides [34].

The initiation of pain assessment at the 6-hour mark was deliberate to ensure that the effects of anesthesia had fully dissipated [35]. The assessment was extended up to 72 h, as it aligns with the timeframe during which the firing of periodontal ligament nociceptive nerves, responsible for postoperative pain, tends to subside after 24 to 48 h [36].

The ability of neurons to regulate inflammatory processes by producing neuropeptides has garnered much attention recently [37]. We measured the amount of SP in our work since it is a neuropeptide that neurons release in response to painful stimuli and causes inflammation [38].

The levels of SP were analyzed from the apical fluid using paper points as they are considered the preferred technique to attain the highest fluid levels at the apical section, even when dealing with minimal amounts of exudates [39]. All paper points carrying apical fluid samples were placed in Eppendorf tubes containing phosphate buffer saline with a 7.4 PH which is non-toxic and can prevent sample cell rupture due to osmosis, ensuring the stability of the sample until further testing [40].

Demographics, including sex and age, showed no significant differences between the two treatment groups. The study’s results revealed that in teeth with SAP, patients in the RG reported significantly higher POP levels at all intervals (6 h, 12 h, 24 h, and 48 h), while both groups showed similar pain level declines at the 72-hour mark. Likewise, SP levels were significantly higher in the RG than in the CRG.

A good explanation for this is the previously documented fact that a higher incidence of pain is greatly attributed to more apical debris extrusion to the periapical area, which mediates an inflammatory response [41, 42] and this extrusion is highly dependent on the type of instrumentation kinematics [43]. A meta-analysis review [44] concluded that the use of rotary instruments in canal preparation is associated with a lower incidence of postendodontic pain than reciprocating instruments due to the reduced debris extrusion caused by the continuous rotation decreasing the irritation and minimizing the inflammation [44].

However, the reciprocating motion involves an initial rotation in a counterclockwise direction, which allows the instrument to penetrate and cut the dentin. Thereafter, a rotation follows in the opposite direction, which makes the flutes unable to remove the debris but rather push them apically [45, 46]. Once more, other studies relate motion kinematics to POP level, concluding that reciprocation kinematics can lead to more POP when compared to continuous rotation [47].

In addition, the absence of reciprocating files #30 and #40 reciprocating files in the sequence of EdgeOne Fire creates higher cutting pressure on the larger reciprocating files, resulting in a greater accumulation of debris aligning with the findings of Nevares et al [48].

Although the current study employed equal volumes of irrigation for both rotary and reciprocating groups, the higher frequency of irrigation cycles applied with the full sequence rotary files effectively reduced the buildup of debris. This, in turn, decreased the likelihood of apical extrusion [49].

Moreover, these results are consistent with the results of studies that compared apically extruded debris with reciprocal and continuous rotational files, concluding greater apical extrusion of debris in reciprocal motion than in continuous rotary motion [50].

In contrast to this study, others reported lower values of postoperative pain with reciprocation than continuous rotation [42, 51, 52] while others showed no significant difference [53, 54].

This inconsistency between studies might be attributed to the differences in the experimental setup, the different file designs, and the subjectivity of pain reported between different patients.

To date, no previous studies have compared the effect of both continuous rotation and reciprocation kinematics on SP in the same study. Caviedes-Bucheli et al. [21] investigated the influence of various rotary instrumentation systems, including ProTaper Universal, RaCe, and Mtwo, on substance P levels. They found that Mtwo exhibited the lowest substance P expression, likely due to its design similarities with EdgeEndo x7, which facilitates efficient debris removal.

### Study limitations

The results need to be validated by additional clinical studies with larger sample sizes because the level of SP in the periapical fluid showed great participant variability. Secondly, patient’s perceptions and reporting of postoperative pain are subjective and differ from one patient to another.

## Conclusion

Continuous rotation motion is accompanied by a lower incidence of postoperative pain and substance P levels when compared to reciprocation motion in patients with symptomatic apical periodontitis.

## Recommendations

From these findings the following recommendations can be made:

- More clinical trials are needed to assess the effect of different instrumentation motions using different files from different manufacturers on SP levels and POP.
- Different inflammatory mediators to be investigated in relation to POP.
- Comparing both continuous rotation and reciprocation motion on different inflammatory mediators in the same study.
- Comparing the mentioned files in this study to files from different manufacturers is recommended to identify the similarities and differences that can affect POP.

## Data Availability

No datasets were generated or analysed during the current study.

## Abbreviations

SP: Substance P
POP: Post Operative Pain
CRG: Continuous Rotation Group
RG: Reciprocation Group
SAP: Symptomatic Apical Periodontitis
NRS: Numerical Rating Scale

## Footnotes

1 R Core Team (2023). R: A language and environment for statistical computing. R Foundation for Statistical Computing, Vienna, Austria. URL https://www.R-project.org/.
